# Rice (*Oryza sativa* L.) roots have iodate reduction activity in response to iodine

**DOI:** 10.3389/fpls.2013.00227

**Published:** 2013-07-10

**Authors:** Shota Kato, Takanori Wachi, Kei Yoshihira, Takuya Nakagawa, Akifumi Ishikawa, Daichi Takagi, Aya Tezuka, Hideharu Yoshida, Satoshi Yoshida, Hitoshi Sekimoto, Michiko Takahashi

**Affiliations:** ^1^Laboratory of Plant Nutrition, Department of Plant Science, United Graduate School of Agricultural Science, Tokyo University of Agriculture and TechnologyUtsunomiya, Japan; ^2^Laboratory of Plant Nutrition, Department of Plant Science, Faculty of Agriculture, Utsunomiya UniversityUtsunomiya, Japan; ^3^Planning and Promotion Unit, Research Center for Radiation Protection, National Institute of Radiological SciencesChiba, Japan

**Keywords:** iodine, reduction, rice, barley, soybean, root

## Abstract

Although iodine is not an essential nutrient for higher plants, their roots take up and transport the element. However, the exact mechanisms involved in iodine uptake and metabolism in higher plants have yet to be elucidated. In this study, we compared two cultivars differing in iodine tolerance (“Nipponbare” and “Gohyakumangoku”) to increasing levels of I^−^ and IO^−^_3_ in the root solutions of water-cultured rice (*Oryza sativa* L.). We found that IO^−^_3_ added to the root solutions was converted to I^−^ in the presence of roots. Iodate reduction occurred over the course of several hours. Furthermore, the iodate reduction activity of “Nipponbare” (iodine-sensitive) and “Gohyakumangoku” (iodine-tolerant) roots increased after adding IO^−^_3_ or I^−^. The roots of barley and soybean also showed iodate reduction activity and the activity responded to iodine treatment either with IO^−^_3_ and I^−^. This study suggests that plant roots biologically reduce iodate to iodide and indicates that the iodate reduction activity of roots responds to external iodine conditions.

## Introduction

Iodine, an essential element for humans, is an important component of thyroid hormones. About 15.8% of the world's population suffers from goiter, which is primarily caused by iodine deficiency. Moreover, an additional one-third of the world's population has been estimated as being at risk of iodine deficiency (De Benoist et al., 2004). One approach to addressing this problem is to increase the iodine content of the edible portions of crops. Recently, iodination of the irrigation waters or fertilizers was investigated to produce iodine-enriched crops (Cao et al., 1994; Weng et al., 2008b; Hong et al., 2009). However, growing of plants with high iodine levels in the irrigation waters is problematic as iodine-toxicity symptoms can develop under these conditions. In addition, inasmuch as iodine is not an essential nutrient for higher plants, detailed mechanisms of iodine uptake and its subsequent metabolism in higher plants have yet to be elucidated.

The major chemical form of soluble iodine in soil solutions is I^−^ under flooded conditions (Muramatsu et al., 1989; Yuita, 1992) and IO^−^_3_ under non-flooded conditions (Yuita, 1992). Iodine uptake and metabolism in plants is dependent on the chemical species present in the irrigation solution. Previous reports have indicated that I^−^ is more phytotoxic than IO^−^_3_ (Umaly and Poel, 1971; Mackowiak and Grossl, 1999; Zhu et al., 2003). Plants have been hypothesized to reduce IO^−^_3_ to I^−^ in culture media (Böszörményi and Cseh, 1960; Muramatsu et al., 1983) and subsequently take up I^−^ from the media (Muramatsu et al., 1983). However, to date, the reduction of IO^−^_3_ and the exact chemical species of iodine taken up by the plant have not been fully established.

In the present study, we investigated any changes in the chemical species of inorganic iodine (I^−^ and IO^−^_3_) in buffer solutions caused by rice root to illuminate the detailed mechanism of iodine uptake by higher plants. Furthermore, we examined the role of rice roots in regard to iodine reduction. From these data, we concluded that iodate reduction is a primary physiological response of rice roots to the presence of iodine.

## Material and methods

### Plant materials

For the test of tolerance to iodine excess, three cultivars of rice were used: *Oryza sativa* L. cv. “Nipponbare,” “Koshihikari” and “Gohyakumangoku.” Rice seeds were sown to the pots with perlite. Rice plants were cultured with the culture media in a green house under natural light condition. Fourteen days-old rice seedlings were subjected to iodine treatment (1 mmol L^−1^ of IO^−^_3_) for 17 days. Composition of the culture media (pH 5.5) was 1.5 mmol L^−1^ NH_4_NO_3_, 1.5 mmol L^−1^ K_2_SO_4_, 0.25 mmol L^−1^ Ca(NO_3_)_2_, 0.5 mmol L^−1^ NH_4_H_2_PO_4_, 1 mmol L^−1^ MgSO_4_ and adequate levels of micronutrients. The media was renewed three times a week. At the end of the iodine treatment, shoot length was measured. For the measurement of iodine concentration in shoots of soil-cultured rice, rice seedlings were subjected to iodine treatment (IO^−^_3_ was mixed with the soil at 1 mmol kg soil^−1^) in the green house under natural light condition. After the IO^−^_3_ treatment, shoots were harvested for the determination of iodine concentration.

For the measurement of iodate reduction activity using rice roots without iodine treatment, rice seedlings (cv. “Koshihikari”) were water-cultured for 27 days in a growth chamber (28/23°C, 12/12 h) with culture solution (pH 5.5) mentioned above. The media were renewed once a week.

For the measurement of iodate reduction activity using rice roots with iodine treatment, water-cultured 21 days-old seedlings (cv. “Nipponbare” and “Gohyakumangoku”) were subjected to iodine treatment for 1 week in a temperature-controlled green house (32/27°C, 12/12 h). I^−^ treatment was performed at 0, 0.025, 0.25, 2.5, 25 μmol L^−1^, and IO^−^_3_ treatment was at 0, 0.25, 2.5, 25, 50, 100 μmol L^−1^. Iodine containing media were renewed twice a week.

For the comparison of iodate reduction activity with barley and soybean roots, water-cultured 10 days-old barley seedlings (*Hordeum vulgare* L. cv. “Mikamogolden”) were subjected to iodine treatment in a temperature-controlled (27/22°C, 12/12 h) green house under natural light condition. Water-cultured 15 days-old soybean seedlings (*Glycine max* cv. “Tachinagaha”) were subjected to iodine treatment in the temperature-controlled (27/22°C, 12/12 h) green house under natural light condition. Twenty-one days-old rice seedlings (cv. “Gohyakumangoku”) were subjected to iodine treatment in the temperature-controlled green house (32/27°C, 12/12 h). I^−^ treatment was performed at 0, 5, 10, 20, 50 μmol L^−1^, and IO^−^_3_ treatment was at 0, 50, 100, 200, 500 μmol L^−1^. Iodine containing media were renewed twice a week after the start of iodine treatment.

### Determination of iodate reduction activity

Rice roots were rinsed with ion-exchanged water and cut at the basal parts. For the determination of iodate reduction activity using roots without iodine treatment, excised roots were immersed in Tris-HCl buffer (50 mmol L^−1^, pH 8.0) containing 0.16 μmol L^−1^ (20 μg L^−1^ as I) of I^−^ or IO^−^_3_. After 24 h of incubation in the dark at 20°C, the buffer solutions were filtered (0.2 μm) and frozen at −80°C immediately. pH of assay buffer was determined to prevent loss of produced I^−^ by automatical change 2 I^−^→I_2_ at lower pH. The concentrations of I^−^ and IO^−^_3_ in the buffer solutions were determined by ion chromatography and inductively coupled plasma-mass spectrometry system (IC-ICP-MS) (Yoshida et al., 2007) described as follows.

For the determination of iodate reduction activity using roots subjected to iodine treatment, excised roots were immersed to Tris-HCl (5 mmol L^−1^, pH 8.0) containing IO^−^_3_ 0.1 mmol L^−1^, followed by 6 h' incubation at 25°C in the dark. After the incubation, the buffer solution was filtered (0.2 μm) and frozen immediately at −80°C until the measurement. Iodate reduction activity was evaluated by the amount of I^−^ reduced from IO^−^_3_ by 1 g (FW) of excised roots per hour. I^−^ concentration in the buffer was determined by 4,4′-methylenebis(*N,N*-dimethylaniline)-chloramine T reaction (Yonehara et al., 1991). To evaluate the net I^−^ concentration reduced from IO^−^_3_ by excised roots, the concentration of I^−^ in the IO^−^_3_-free buffer incubated with excised roots was also determined as I^−^ derived from excised roots during incubation, and subtracted from I^−^ concentration in the IO^−^_3_ buffer incubated with excised roots.

### The separate determination of concentration of I^−^ and IO^−^_3_ in the buffer solutions

The concentrations of I^−^ and IO^−^_3_ in the buffer solutions were determined by ion chromatography (IC: IC7000S, Column: EXCELPAK ICS-A23, Yokogawa Analytical Systems Inc.) and inductively coupled plasma-mass spectrometry (7500, Agilent) system (IC-ICP-MS) (Yoshida et al., 2007). The detection limit of the system was 8 nmol L^−1^ of I^−^ and IO^−^_3_.

### Determination of total iodine concentration in plant tissue

About 0.1 g of plant samples (shoots and roots) dried at 80°C were digested by 25% tetramethyl ammonium hydroxide (TAMAPURE-AA TMAH, Tama Chemicals Co., Ltd.) in a 6 ml PFA vial (Savillex Co.) overnight at 80°C (Tagami et al., 2006). Concentration of ^127^I in the solution was determined by ICP-MS (7500, Agilent).

## Results

### Tolerance among three rice cultivars to high solution levels of iodine

**Figure 1:**
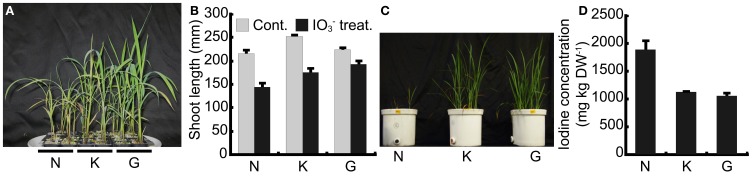
**Tolerance among three rice cultivars, “Nipponbare” (N), “Koshihikari” (K), and “Gohyakumangoku” (G) to iodine excess. (A)** Rice plants treated with IO^−^_3_. Rice plants were cultured in pots filled with perlite and Kasugai's nutrient solution (pH 5.5) containing IO^−^_3_. **(B)** Shoot length of rice plants treated with or without IO^−^_3_. Cont.: rice plants treated without IO^−^_3_, IO^−^_3_ treat.: rice plants treated with IO^−^_3_. Data are mean ± standard error (*n* = 6). **(C)** Shoots of soil-cultured rice cultivars after the iodine treatment. Rice seedlings were grown in 1/5000a Wagner pots with soil mixed with IO^−^_3_. **(D)** Concentration of iodine in the shoots of soil-cultured rice **(C)** after the IO^−^_3_ treatment. Data are mean ± standard error (*n* = 4).

To confirm the tolerance levels of our three rice cultivars, “Nipponbare,” “Koshihikari” and “Gohyakumangoku” to high iodine concentrations, we treated each cultivar with 1 mmol L^−1^ IO^−^_3_. Figure 1A shows plants treated with IO^−^_3_ in the pots filled with perlite and culture media. Visible symptoms of iodine toxicity were observed in all three cultivars. The basal part of the stem developed a reddish-brown color. In addition, reddish-brown puncta were observed in the lower leaves. The symptoms were the most visible in “Nipponbare” and the least apparent in “Gohyakumangoku.” Chlorosis was also observed in leaves of both “Nipponbare” and “Gohyakumangoku.” Figure 1B shows the shoot length of the three rice cultivars treated with or without IO^−^_3_ for 17 days. In our IO^−^_3_ treatment, the average shoot lengths of “Nipponbare,” “Koshihikari” and “Gohyakumangoku” were 145, 176, and 193 mm, respectively.

**Figure A1:**
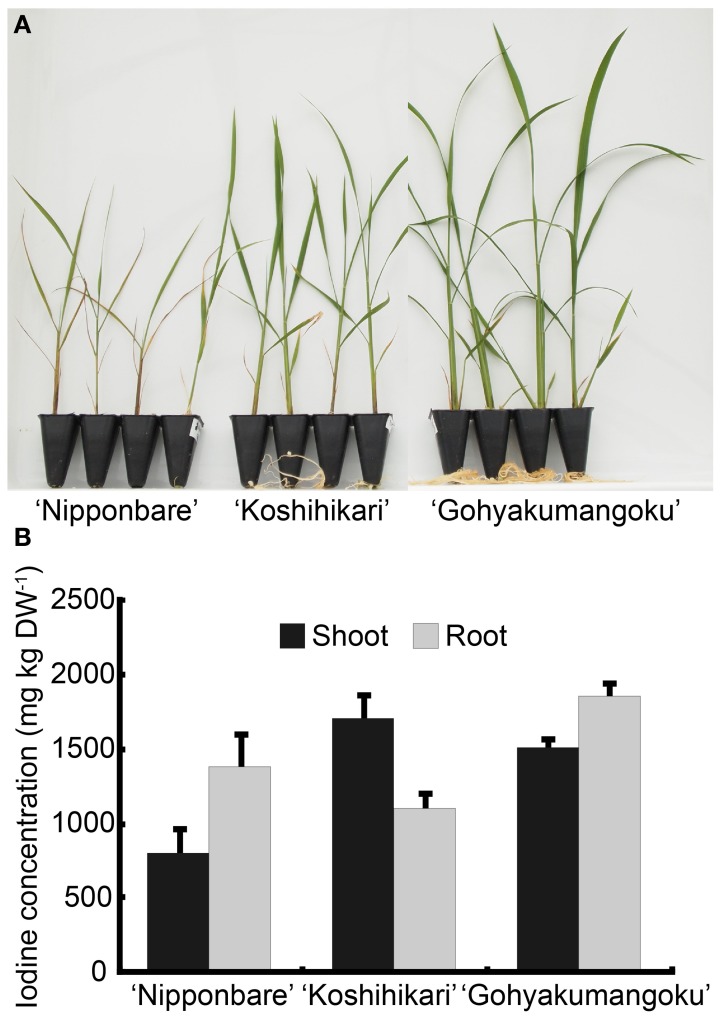
**(A)** Rice plants of three rice cultivars (cv. “Nipponbare,” “Koshihikari,” and “Gohyakumangoku”) after the I^−^ treatment in the pots filled with perlite. Seedlings were cultured with Kasugai's nutrient solution (pH 5.5) in the green house under natural light condition. Twelve days-old rice seedings were subjected to iodine treatment (I^−^ 100 μmol L^−1^) for 21 days. **(B)** Concentration of iodine in shoots and roots of rice (“Nipponbare,” “Koshihikari,” and “Gohyakumangoku”) after the I^−^ treatment. Data are mean ± standard error (*n* = 4).

Figure 1C illustrates the tolerance of levels among three cultivars grown with IO^−^_3_-mixed soil under flooded conditions. Growth retardation by iodine treatment was most apparent in “Nipponbare.” Iodine concentration in shoots of “Nipponbare” was 1.7 and 1.8 times as much as that of “Koshihikari” and “Gohyakumangoku,” respectively (Figure 1D). The tolerance levels among the three cultivars to excess iodine in I^−^ treatment were similar to those in IO^−^_3_ treatment (Figure A1A). Based on these results, “Nipponbare” was used as our iodine-sensitive cultivar and “Gohyakumangoku” as our iodine-tolerant cultivar in this study.

### Iodate reduction by rice roots

**Figure 2:**
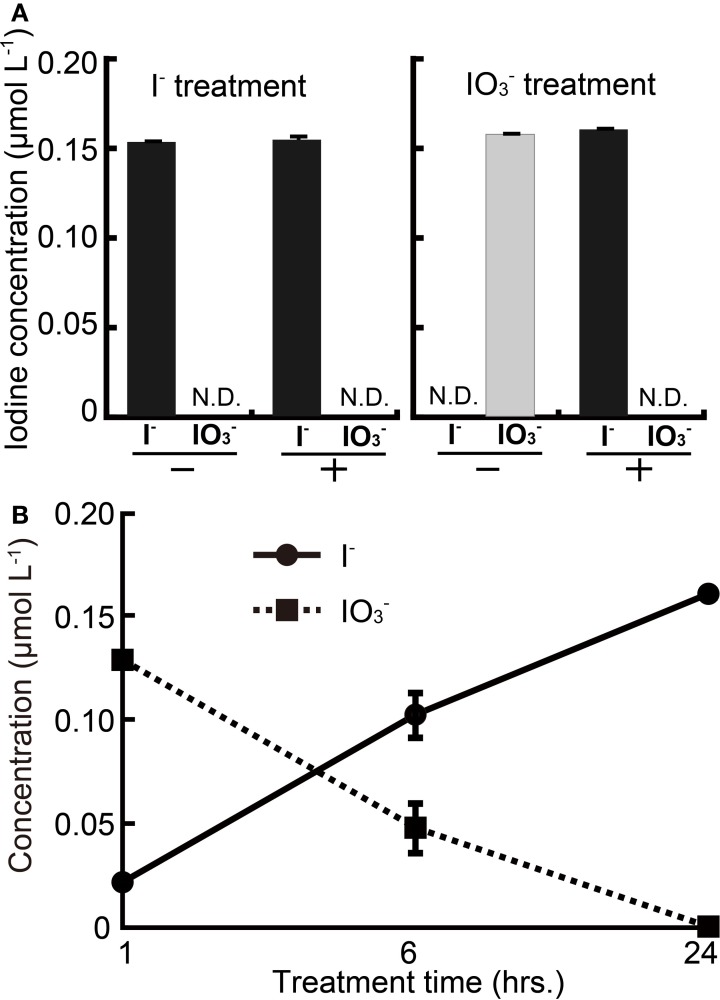
**(A)** Changes in concentration of I^−^ or IO^−^_3_ after the 24 h' incubation with or without rice roots. I^−^ treatment: incubation using the buffer containing I^−^, IO^−^_3_ treatment: incubation using the buffer containing IO^−^_3_, +: incubation with roots, −: incubation without roots. (**B)** Time course analysis of I^−^ and IO^−^_3_ concentration in IO^−^_3_ solution with excised rice roots. Excised rice roots were immersed to the incubation buffer containing IO^−^_3_ for 1, 6, and 24 h. Data are mean ± standard error (*n* = 3).

To investigate any changes in chemical species of iodine caused by rice roots, excised rice roots were immersed in solutions of I^−^ or IO^−^_3_. The roots of “Koshihikari” were used because it showed medium-tolerance to iodine excess between “Nipponbare” and “Gohyakumangoku.” Figure 2A shows the concentrations of I^−^ or IO^−^_3_ in the solutions of I^−^ or IO^−^_3_ after a 24-h incubation period with or without root tissue. The concentrations of I^−^ or IO^−^_3_ were not changed in the solutions of I^−^ or IO^−^_3_ without roots after the incubation. The concentration of I^−^ was also unchanged in the I^−^ solution with roots after the incubation. On the other hand, the concentration of I^−^ was increased in the IO^−^_3_ solution with roots after the incubation.

A time course of changes in chemical species of iodine was also analyzed in the solutions of IO^−^_3_ with roots (Figure 2B). The concentration of IO^−^_3_ in IO^−^_3_ solution was decreased over 24 h. Meanwhile, I^−^ concentrations in IO^−^_3_ solution were increased over the same time period. This result indicated that the chemical form of iodine was changed between I^−^ and IO^−^_3_ and suggested that almost all the IO^−^_3_ in the solution were reduced to I^−^ by rice roots.

### Effect of iodine treatment on the growth of an iodine-sensitive and an iodine-tolerant cultivar

**Figure 3:**
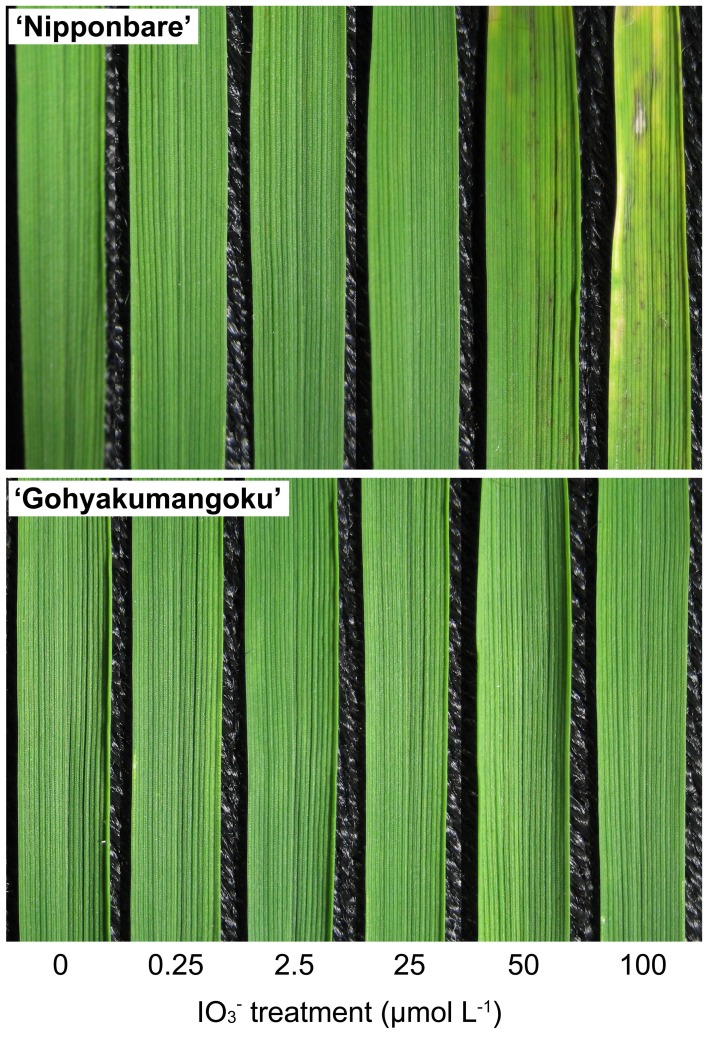
**The third leaves of “Nipponbare” and “Gohyakumangoku” after the IO^−^_3_ treatment at varying concentration**. Rice seedling were treated with IO^−^_3_ at 0, 0.25, 2.5, 25, 50, 100 μmol L^−1^.

Figure 3 shows the third leaves of “Nipponbare” and “Gohyakumangoku” 1 week after IO^−^_3_ treatment at increasing iodine concentrations. At the iodine concentration below 25 μmol L^−1^ IO^−^_3_, visible iodine-toxicity symptoms were not observed in the shoots of “Nipponbare.” However, mild chlorosis and reddish-brown puncta were observed in the shoot of “Nipponbare” at 50 μmol L^−1^ IO^−^_3_, and severe symptoms at 100 μmol L^−1^ IO^−^_3_. Conversely, visible changes were not observed in the shoots of “Gohyakumangoku” at all concentrations of the IO^−^_3_ treatment.

Table 1 shows the length and fresh weight of both the shoots and roots of “Nipponbare” and “Gohyakumangoku” after IO^−^_3_ treatment. The shoot length of “Nipponbare” was decreased in our IO^−^_3_ treatment in a concentration-dependent manner. The shoot length of “Nipponbare” at 100 μmol L^−1^ IO^−^_3_ was 85% that of the control. Significant changes were not observed in shoot fresh weight, root length, or root fresh weight of “Nipponbare” by IO^−^_3_ treatment. The length and fresh weight of the shoot and root of “Gohyakumangoku” were not significantly affected by IO^−^_3_ treatment.

**Table 1:** **Effect of the IO^−^_3_ treatment on length and fresh weight of shoots and roots of A “Nipponbare” and B “Gohyakumangoku”**.

**IO^−^_3_ treatment (μmol L^−1^)**	**Length (mm)**		**Fresh weight (g)**	
	**Shoot**	**Root**	**Shoot**	**Root**
**(A)**				
0	292 ± 5^a,b^	116 ± 8^a^	1.00 ± 0.06^a,b^	0.28 ± 0.02^a^
0.25	290 ± 12^a,b^	112 ± 4^a^	1.06 ± 0.07^a,b^	0.29 ± 0.03^a^
2.5	301 ± 3^a^	106 ± 5^a^	1.09 ± 0.06^a^	0.29 ± 0.02^a^
25	287 ± 7^a,b^	114 ± 2^a^	1.04 ± 0.02^a,b^	0.30 ± 0.01^a^
50	260 ± 11^b,c^	120 ± 4^a^	0.84 ± 0.04^b^	0.25 ± 0.01^a^
100	249 ± 4^c^	122 ± 2^a^	0.92 ± 0.05^a,b^	0.25 ± 0.01^a^
**(B)**				
0	297 ± 8^a^	142 ± 5^a^	1.27 ± 0.07^a^	0.37 ± 0.02^a^
0.25	297 ± 3^a^	131 ± 2^a^	1.21 ± 0.07^a^	0.32 ± 0.02^a^
2.5	318 ± 16^a^	132 ± 5^a^	1.23 ± 0.03^a^	0.37 ± 0.01^a^
25	308 ± 2^a^	138 ± 4^a^	1.18 ± 0.04^a^	0.34 ± 0.01^a^
50	305 ± 2^a^	133 ± 5^a^	1.27 ± 0.07^a^	0.41 ± 0.03^a^
100	302 ± 3^a^	144 ± 4^a^	1.19 ± 0.03^a^	0.37 ± 0.02^a^

Data in the columns are mean ± standard error (n = 5). Values in each column followed by the same letter are not significantly different (Tukey's multiple range test, P < 0.05).

**Figure 4:**
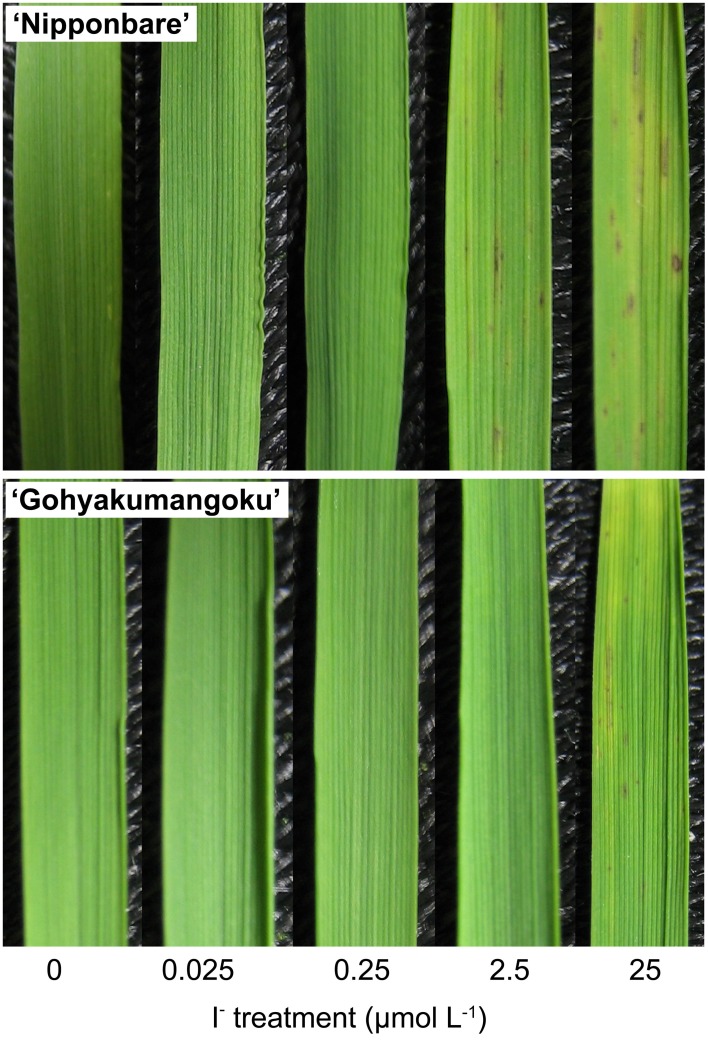
**The third leaves of “Nipponbare” and “Gohyakumangoku” after the I^−^ treatment at varying concentration**. Rice seedlings were treated with I^−^ at 0, 0.025, 0.25, 2.5, 25 μmol L^−1^.

The third leaves of “Nipponbare” and “Gohyakumangoku” plants subjected to varying I^−^ concentrations for 7 days are shown in Figure 4. In the leaves of “Nipponbare,” mild and severe iodine-toxicity symptoms appeared in our 2.5 and 25 μmol L^−1^ I^−^ treatments, respectively. When subjected to 25 μmol L^−1^ of I^−^ treatment, the leaves of “Gohyakumangoku” plants also exhibited visible iodine-toxicity symptoms.

The growth of “Nipponbare” shoots decreased in the plants grown under I^−^ treatment. This decrease was also in a concentration-dependent manner (Table 2A). In our 2.5 and 25 μmol L^−1^ treatments, we measured a 20% and 26% decrease, respectively, in shoot length compared to the controls. The shoot fresh weight was only 70% and 58% that of the controls at 2.5 and 25 μmol L^−1^ I^−^, respectively. In the case of “Nipponbare” root growth, the fresh weight of the roots under 2.5 and 25 μmol L^−1^ I^−^ treatment decreased to 68 and 58%, respectively, of the control value. Meanwhile, root length did not appear to be significantly affected by I^−^ treatment. With respect to our tolerant cultivar, we found no significant effects on length and fresh weight by I^−^ treatment in both shoot and roots (Table 2B).

**Table 2:** **Effect of the I^−^ treatment on length and fresh weight of shoots and roots of A “Nipponbare” and B “ Gohyakumangoku”**.

**I^−^ treatment (μmol L^−1^)**	**Length (mm)**		**Fresh weight (g)**	
	**Shoot**	**Root**	**Shoot**	**Root**
**(A)**				
0	261 ± 12^a^	137 ± 8^a^	0.89 ± 0.04^a^	0.40 ± 0.02^a^
0.025	255 ± 7^a^	145 ± 14^a^	0.87 ± 0.03^a^	0.37 ± 0.01^a,b^
0.25	235 ± 4^a,b^	135 ± 11^a^	0.83 ± 0.05^a^	0.33 ± 0.01^b^
2.5	209 ± 3^b,c^	112 ± 6^a^	0.62 ± 0.02^b^	0.27 ± 0.01^c^
25	193 ± 3^c^	131 ± 7^a^	0.52 ± 0.03^b^	0.23 ± 0.01^c^
**(B)**				
0	219 ± 5^a^	110 ± 2^a,b^	0.70 ± 0.02^a–c^	0.32 ± 0.01^a^
0.025	223 ± 4^a^	111 ± 3^a,b^	0.72 ± 0.02^a,b^	0.31 ± 0.00^a^
0.25	239 ± 9^a^	108 ± 3^a,b^	0.76 ± 0.02^a^	0.33 ± 0.01^a^
2.5	230 ± 6^a^	124 ± 6^a^	0.66 ± 0.02^b,c^	0.30 ± 0.00^a^
25	237 ± 5^a^	105 ± 3^b^	0.62 ± 0.03^c^	0.30 ± 0.01^a^

Data in the columns are mean ± standard error (n = 6). Values in each column followed by the same letter are not significantly different (Tukey's multiple range test, P < 0.05).

### Effect of iodine treatments on iodate reduction activity in root tissue

To investigate the effect of iodine in rhizosphere on iodate reduction activity in root tissue, iodate reduction activity was measured after treatment with two kinds of species of iodine, IO^−^_3_ and I^−^, at varying concentrations.

**Figure 5:**
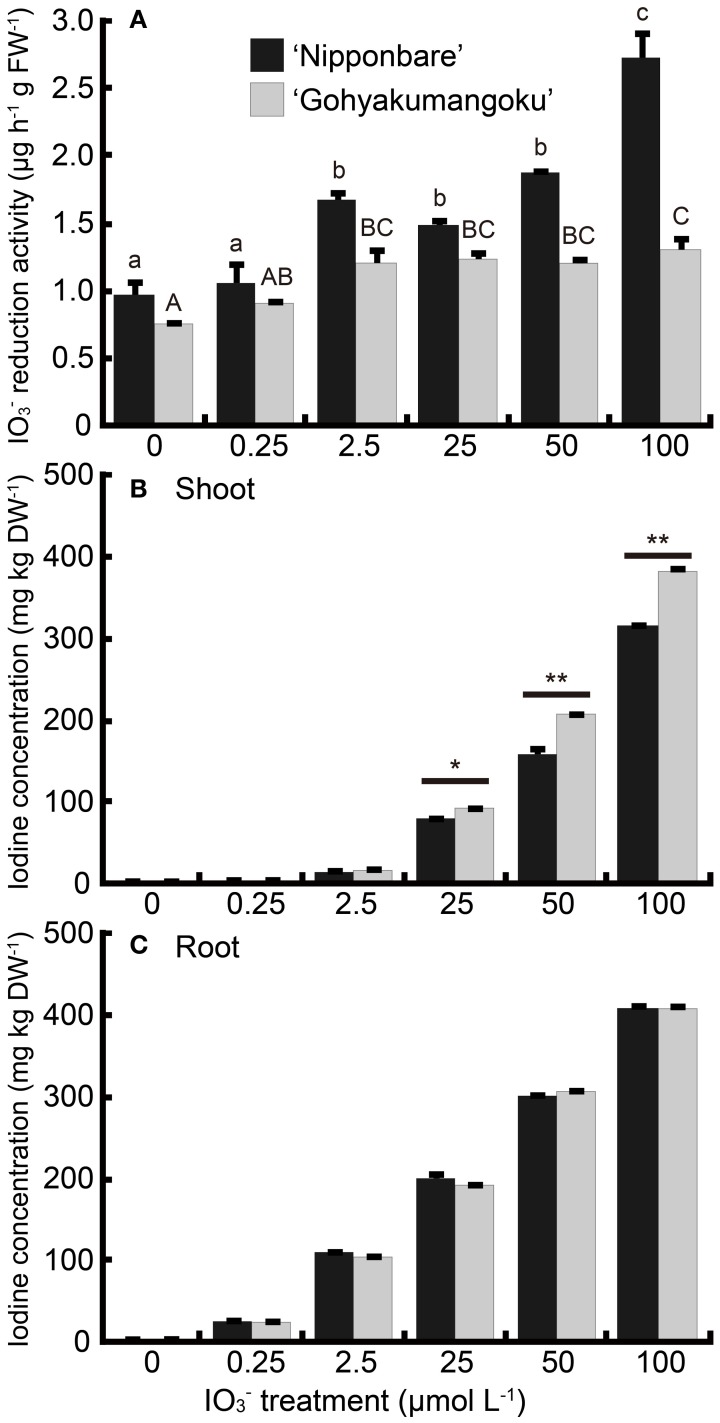
**(A)** Effect of the IO^−^_3_ treatment on iodate reduction activity of excised rice roots. After the IO^−^_3_ treatment at 0, 0.25, 2.5, 25, 50, 100 μmol L^−1^, iodate reduction activity (the amount of I^−^ reduced from IO^−^_3_ by 1 g (FW) of excised roots per hour) was measured. Data are mean ± standard error (*n* = 3). Bars with the same letter are not significantly different among each iodine treatments in each cultivar (Tukey's multiple range test, *P* < 0.05). **(B, C)** Concentration of total iodine in **(B)** shoots and **(C)** roots of “Nipponbare” and “Gohyakumangoku” after the IO^−^_3_ treatment. Data are mean ± standard error (*n* = 3). ^*^P < 0.05; ^**^P < 0.01, *t*-test.

Figure 5A shows the iodate reduction activity of roots of “Nipponbare” and “Gohyakumangoku” treated with IO^−^_3_ for 7 days at increasing concentrations. Iodate reduction by the roots was higher in “Nipponbare” than in “Gohyakumangoku” at all concentrations tested. Iodate reduction increased in “Nipponbare” treated with IO^−^_3_. Iodate reduction increased in a concentration-dependent manner, even at concentrations resulting in the appearance of symptoms indicating iodine toxicity. Iodate reduction activity increased up to 2.8-fold at 100 μmol L^−1^ IO^−^_3_. On the other hand, root iodate reduction activity did not increase at concentrations higher than 2.5 μmol L^−1^ IO^−^_3_ in the “Gohyakumangoku” cultivar. However, at 2.5 μmol L^−1^ IO^−^_3_, reduction activity increased 1.6-fold.

Iodine concentrations in both the shoots and roots of “Nipponbare” and “Gohyakumangoku” after IO^−^_3_ treatment are shown in Figures 5B, C, respectively. The concentrations of iodine in the shoots and roots of both cultivars increased with IO^−^_3_ treatment in a concentration-dependent manner. The difference in the shoot iodine concentrations between the two cultivars was magnified at the highest iodine treatment. The shoot iodine concentrations were higher in the shoots of “Gohyakumangoku.” Unexpectedly, we found no significant difference in iodine concentration in the roots of our two cultivars.

**Figure 6:**
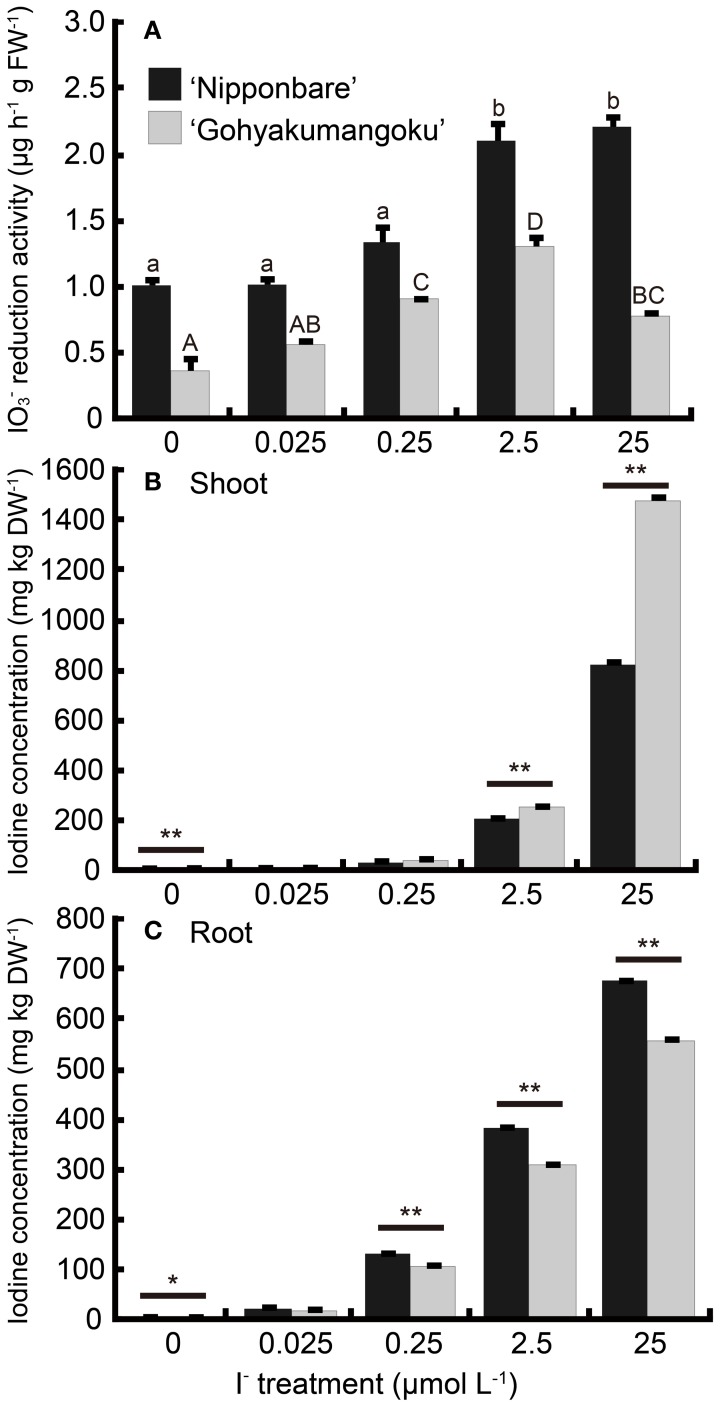
**(A)** Effect of the I^−^ treatment on iodate reduction activity of excised rice roots. After the I^−^ treatment at 0, 0.025, 0.25, 2.5, 25 μmol L^−1^, iodate reduction activity was measured. Data are mean ± standard error (*n* = 3). Bars with the same letter are not significantly different among each iodine treatments in each cultivar (Tukey's multiple range test, *P* < 0.05). **(B, C)** Concentration of total iodine in **(B)** shoots and **(C)** roots of “Nipponbare” and “Gohyakumangoku” after the I^−^ treatment. Data are mean ± standard error (*n* = 3). ^*^P < 0.05; ^**^P < 0.01, *t*-test.

Root iodate reduction activities of rice treated with I^−^ are shown in Figure 6A. Root iodate reduction was higher in “Nipponbare” at all concentrations tested. Even at concentrations resulting in iodine-toxicity symptoms, we found that root iodate reduction increased twofold in “Nipponbare.” Our tolerant cultivar, “Gohyakumangoku” also demonstrated an increase in reduction activity due to I^−^ treatment in a concentration-dependent manner. However, reduction activity decreased at treatment levels resulting in toxicity symptoms.

Figures 6B, C shows the total iodine concentrations in both the shoot and roots of rice plants after 7 days of I^−^ treatment. The concentrations of iodine in shoots and roots of both cultivars increased in a concentration-dependent manner with I^−^ treatment. Also, we found that the iodine levels were higher in the shoots and lower in the roots of “Gohyakumangoku” than those of “Nipponbare.” The difference in the concentration levels in these tissues between the two cultivars increased in a concentration-dependent manner with I^−^ treatment. At 25 μmol L^−1^ I^−^, the iodine concentrations in the shoots of “Gohyakumangoku” was 1.8 times greater than that of “Nipponbare.”

**Figure 7:**
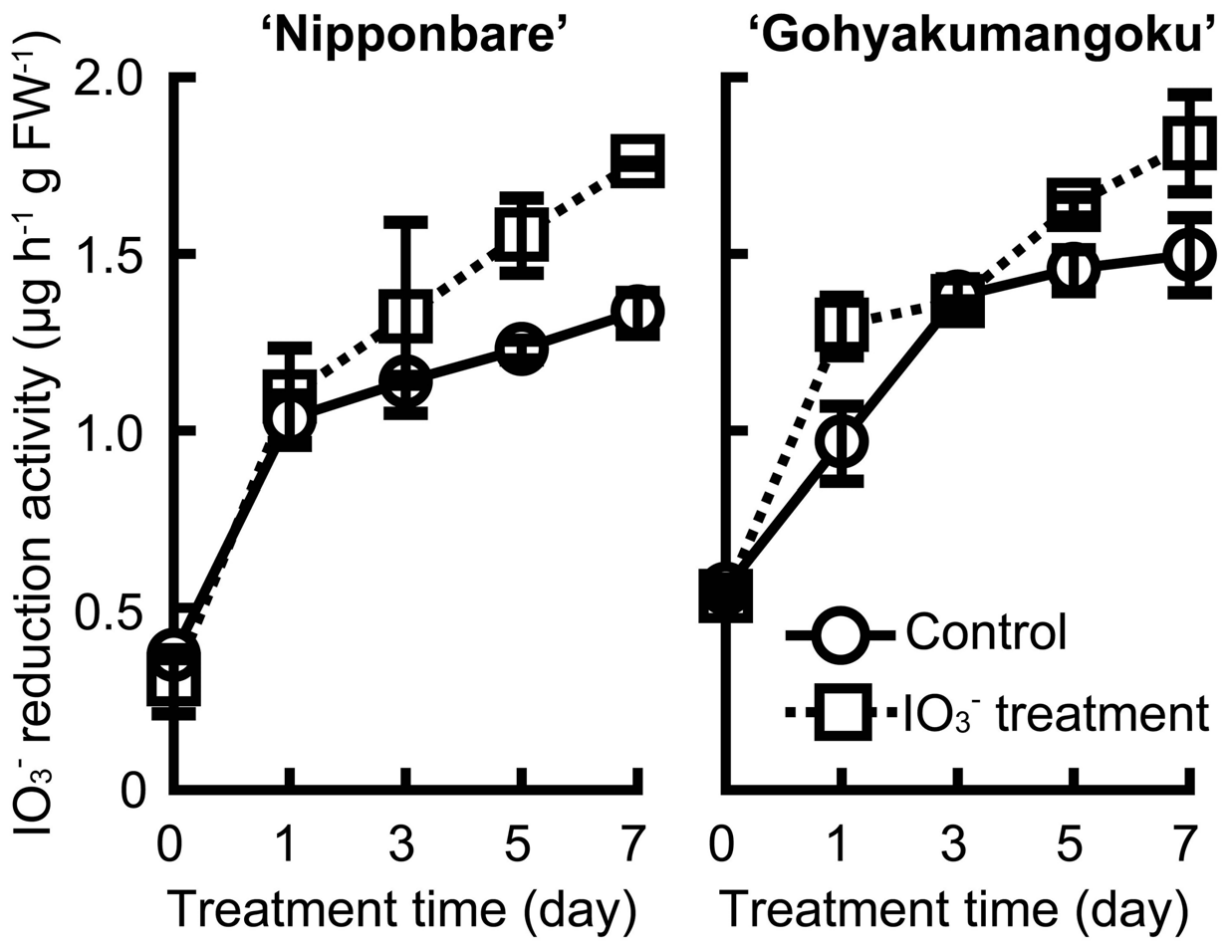
**Effect of the IO^−^_3_ treatment time on iodate reduction activity of excised rice roots**. Rice seedlings (cv. “Nipponbare” and “Gohyakumangoku”) were subjected to 0 and 100 μmol L^−1^ of IO^−^_3_ for 0, 1, 3, 5, and 7 days. After the IO^−^_3_ treatment, iodate reduction activity was measured. Data are mean ± range (*n* = 2) Control: iodate reduction activity of rice roots treated without IO^−^_3_, IO^−^_3_ treatment: iodate reduction activity of rice roots treated with IO^−^_3_.

Figure 7 shows the iodate reduction activity of roots of “Nipponbare” and “Gohyakumangoku” treated with 0 and 100 μmol L^−1^ of IO^−^_3_ for 7 days. Iodate reduction activity of roots was increased with age, and induced apparently by 7 days' IO^−^_3_ treatment.

### Iodate reduction activity in root tissue of barley and soybean

**Figure 8:**
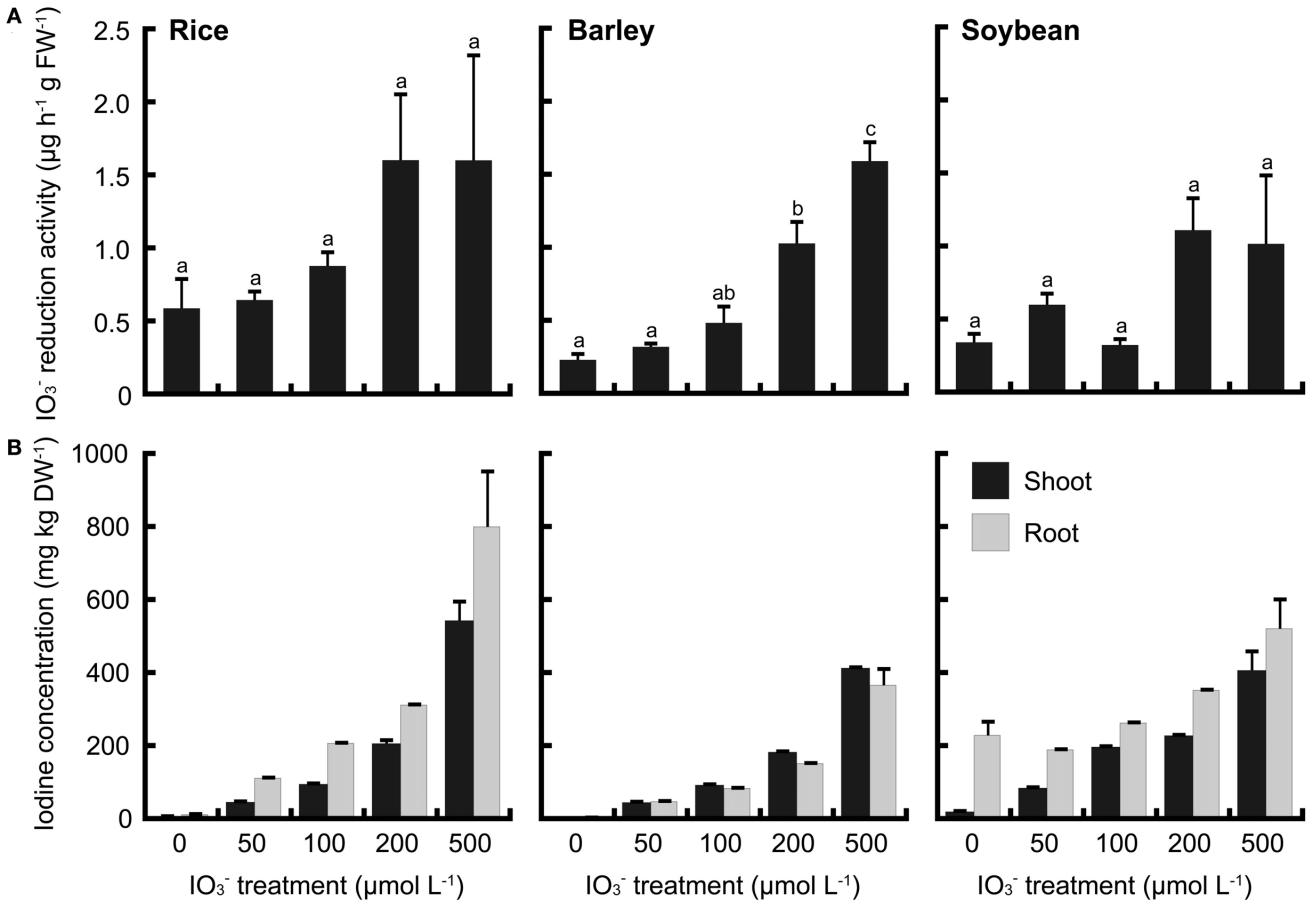
**(A)** Effect of the IO^−^_3_ treatment on iodate reduction activity of excised roots in rice, barley and soybean. After the IO^−^_3_ treatment at 0, 50, 100, 200, and 500 μmol L^−1^, iodate reduction activity was measured. Data are mean ± standard error (*n* = 3). Bars with the same letter are not significantly different among each iodine treatments (Tukey's multiple range test, *P* < 0.05). **(B)** Concentration of total iodine in shoots and roots of rice, barley and soybean after the IO^−^_3_ treatment. Data are mean ± standard error (*n* = 3).

**Figure 9:**
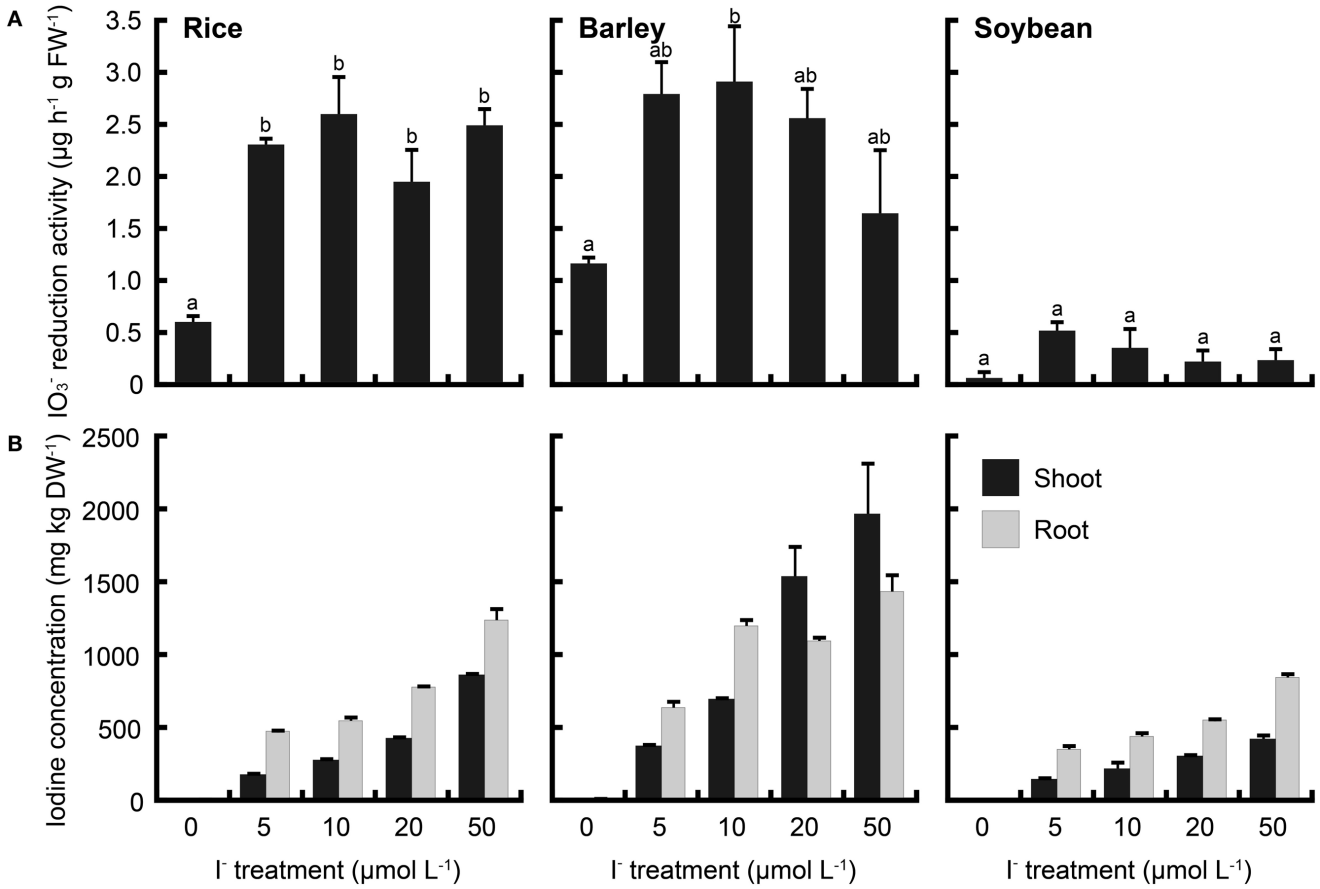
**(A)** Effect of the I^−^ treatment on iodate reduction activity of excised roots in rice, barley and soybean. After the I^−^ treatment at 0, 5, 10, 20, and 50 μmol L^−1^, iodate reduction activity was measured. Data are mean ± standard error (rice and soybean; *n* = 3, barley; *n* = 6). Bars with the same letter are not significantly different among each iodine treatments (Tukey's multiple range test, *P* < 0.05). **(B)** Concentration of total iodine in shoots and roots of rice, barley and soybean after the I^−^ treatment. Data are mean ± standard error (*n* = 3).

To compare these phenomena of rice with those of upland crops, iodate reduction activity of roots was investigated in barley and soybean. Barley and soybean showed also iodate reduction activity (Figures 8A, 9A). In IO^−^_3_ treatment, iodate reduction activity was the highest in rice among these three plant species (Figure 8A). Iodine concentrations in the shoots and roots of rice were higher than those of barley and soybean (Figure 8B). In I^−^ treatment, the iodate reduction activity and iodine concentrations in the shoots and roots were the highest in barley, and the lowest in soybean (Figure 9).

## Discussion

Rice plants often display iodine-toxicity symptoms, known as *reclamation Akagare disease* or Akagare type III, when grown in upland fields that have been converted to lowland fields in some volcanic ash soils (Baba et al., 1964; Tensho, 1970; Watanabe and Tensho, 1970). In soil solutions, the major chemical species of soluble iodine are generally thought to be I^−^ under flooded conditions (Muramatsu et al., 1989; Yuita, 1992) and IO^−^_3_ under non-flooded conditions (Yuita, 1992). At similar concentrations, I^−^ appears to be more phytotoxic than IO^−^_3_ (Umaly and Poel, 1971; Mackowiak and Grossl, 1999; Zhu et al., 2003; Weng et al., 2008a). Böszörményi and Cseh (1960) suggested that IO^−^_3_ is reduced to I^−^ electrochemically before uptake by wheat roots. Muramatsu et al. (1983) reported that the Komatsuna plant, *Brassica rapa* var. *pervidis*, can accelerate the conversion of IO^−^_3_ to I^−^ in culture solution. Together, these reports indicate that in culture media, plants can reduce IO^−^_3_ to I^−^ and would take up I^−^. Recently, Yamada et al. (2005) further proposed that rice roots might even take up I_2_ oxidized from I^−^.

To date, however, whether plant roots physiologically function to change the chemical species of iodine in the rhizosphere remains unclear. To address this question, we performed a set of experiments aimed at investigating the role, if any, of rice roots in changing the chemical species of iodine. Additionally, we examined any changes occurring in the iodate reduction activity of roots by the presence of external iodine.

### Differences in iodine tolerance in rice

To confirm iodine tolerance among rice cultivars, we selected three rice cultivars and cultured them under the excess condition of two forms of iodine, i.e., iodide (I^−^) and iodate (IO^−^_3_). Our results clearly demonstrate the degree of tolerance to excess iodine (I^−^ and IO^−^_3_) among these cultivars: “Nipponbare” < “Koshihikari” < “Gohyakumangoku” (Figures 1, A1). These results are in agreement with those reported by Yamada et al. (2006). However, it was considered that the tolerance levels among three rice cultivars to excess iodine had no obvious relation to iodine concentration in shoots as described below.

### Iodate reduction by root tissue

To elucidate whether rice roots convert IO^−^_3_ or I^−^ to some other chemical species of iodine, concentrations of IO^−^_3_ or I^−^ were determined in iodine solutions incubated with the excised roots of “Koshihikari.” Almost all of IO^−^_3_ added to the buffer was reduced to I^−^ by the excised roots during a 24-h incubation period. However, the concentration of I^−^ was unchanged in the I^−^ solution with roots after the incubation (Figure 2A). In addition, our time-course analysis indicated that iodate reduction occurred over the course of several hours (Figure 2B). Collectively, these results indicate that rice roots have the ability to reduce IO^−^_3_ to I^−^. Earlier, a report stated that when grown at similar iodine levels, I^−^-treated plants had a higher iodine content than IO^−^_3_-treated plants (Muramatsu et al., 1983; Mackowiak and Grossl, 1999; Zhu et al., 2003; Weng et al., 2008a; Voogt et al., 2010). Our results suggest that plants would take up I^−^ reduced from IO^−^_3_.

### Effect of iodine excess on rice shoots and roots

From our observations, the concentration of iodine in our I^−^ treatment that caused adverse effects on growth of both the sensitive and tolerant cultivars was much lower than that of our IO^−^_3_ treatment (Tables 1, 2). This result is consistent with previous reports in the literature (Umaly and Poel, 1971; Mackowiak and Grossl, 1999; Zhu et al., 2003; Weng et al., 2008a). Plant height decreased with high iodine treatment in both the soil culture and water culture (Figures 1A–C, A1A, Tables 1, 2). On the other hand, root length was not significantly affected, although root fresh weight decreased under the high iodine treatment, especially I^−^ excess. This may in part have been due to the age of the treated plants. Also, the emergence of new roots appeared to be suppressed at high iodine levels in the growth solutions. Decrease in root fresh weight is related to the inhibition of elongation of new roots. High iodine levels would also inhibit the elongation of roots as with any other excessive nutrient condition.

### Iodine concentration in plant body and tolerance to iodine excess

Under the water-cultured condition, iodine concentration in the shoots of “Gohyakumangoku” (tolerant) was higher than that of “Nipponbare” (sensitive) in both the IO^−^_3_ and I^−^ treatments (Figures 5B, 6B, A1B). On the other hand, under the soil-cultured condition, iodine concentration in the shoots of “Gohyakumangoku” (tolerant) was lower than that of “Nipponbare” (sensitive) in the IO^−^_3_ treatments (Figure 1D). However, “Gohyakumangoku” showed higher tolerance to excess iodine than “Nipponbare” regardless of iodine concentration in shoots under both conditions. Yamada et al. (2006) also reported that iodine concentration in shoots of “Gohyakumangoku” was much lower than that of “Nipponbare” under soil culture. The discrepancy between these results was considered due to the different culture condition employed, namely soil-cultured (Figure 1D) or water-cultured (Figure 5B). Therefore, it is considered that the tolerance levels among three rice cultivars to excess iodine had no obvious relation to iodine concentration in shoots. In our study, higher iodine concentration in “Gohyakumangoku” (tolerant) shoots suggests that the form or localization of iodine within the plant body is more important than the total concentration of iodine. The higher tolerance of “Gohyakumangoku” to iodine stress might correlate with the species of iodine transported or stored within the plant body (e.g., vacuole and intercellular space).

### Induction of iodate reduction activity in response to external iodine

Iodate reduction activity displayed by “Nipponbare” and “Gohyakumangoku” roots increased with either IO^−^_3_ or I^−^ treatment. In “Gohyakumangoku,” iodate reduction activity decreased at concentrations of iodine that resulted in visible iodine-toxicity symptoms (Figures 5A, 6A).

The induction of iodate reduction by I^−^ treatment was an unexpected phenomenon since our original hypothesis predicted that plants would take up I^−^ reduced from IO^−^_3_. The mechanism of this induction is still unclear. Unidentified oxidation of I^−^ might be related to this induction since the oxidation activity of roots detected by ∂-naphtylamine was also increased by iodine treatment (data not shown).

The decrease in iodate reduction under high iodine conditions seems to correlate with iodine tolerance. Because lower iodate reduction activity will be helpful under excess iodine condition since high iodate reduction activity can contribute to uptake of I^−^, more toxic form of iodine. Lower concentration of iodine in roots of “Gohyakumangoku” might be related to lower iodate reduction activity in I^−^ treatment. Further detailed studies are needed to fully clarify these responses.

However, our results suggest that iodate reduction in rice roots is related, at least in part, to external iodine conditions. The induction of iodate reduction activity in IO^−^_3_ treatment was suppressed by removing iodine from culture media (data not shown). We also demonstrated that barley and soybean roots converted IO^−^_3_ to I^−^, and that iodate reduction activity of barley and soybean roots increased with both IO^−^_3_ and I^−^ treatment (Figures 8, 9).

### The mechanism of uptake and transport of iodine in plants

**Figure 10:**
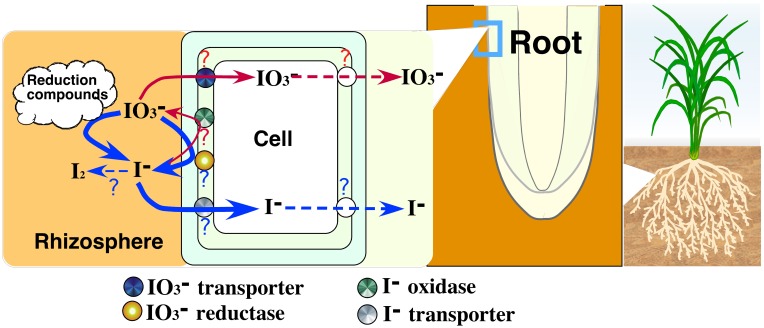
**The model diagram showing our hypothesis of uptake of iodine by higher plants**.

This is the first report demonstrating that plant roots have an ability to reduce IO^−^_3_ to I^−^ and that iodate reduction activity in roots could respond to external iodine. We show our hypothesis of uptake of iodine by roots in Figure 10. Conversion of IO^−^_3_ to I^−^ by the excised roots suggests the existence of iodate reductase in roots. Iodate would be reduced by iodate reduction compounds released from the roots treated with iodine (data not shown). Under excess iodine condition, it is considered that I^−^ is mainly absorbed by roots due to high iodate reduction activity of iodate reductase. The existence of iodate reductase induced by iodine would be suggested by the induction of iodate reduction activity of crude proteins extracted from rice roots treated with iodine (data not shown). Iodide oxidase might also exist and be related to the iodate reduction in I^−^ treatment. The uptake of iodine could be regulated by transporters in plasma membrane. Transport of iodine from roots to shoots could be also regulated by transporters. In barley treated with higher I^−^, iodine concentration in shoots reversed that of roots. In IO^−^_3_ treatment, iodine concentration in shoots was higher than that of roots only in barley. These results suggest the existence of iodine transporters. The major chemical form of soluble iodine in soil solutions is I^−^ under flooded conditions (Muramatsu et al., 1989; Yuita, 1992) and IO^−^_3_ under non-flooded conditions (Yuita, 1992). Soybean and barley showed the difference in the iodate reduction activity and iodine concentrations in plant body between in I^−^ and IO^−^_3_ treatment. This might suggest the difference in the mechanism of uptake and transport of iodine between paddy-rice and upland crops.

### Conflict of interest statement

The authors declare that the research was conducted in the absence of any commercial or financial relationships that could be construed as a potential conflict of interest.

## References

[B1] Baba I. Inada K. Tajima K. (1964). Mineral nutrition and the occurrence of physiological diseases, in The Mineral Nutrition of the Rice Plant, ed IRRI (Baltimore, MD: The Johns Hopkins Press), 173–195

[B2] Böszörményi Z. Cseh E. (1960). The uptake and reduction of iodate by wheat-roots. Curr. Sci. 29, 340–341

[B3] Cao X. Y. Jiang X. M. Kareem A. Dou Z. H. Abdul Rakeman M. Zhang M. L. (1994). Iodination of irrigation water as a method of supplying iodine to a severely iodine-deficient population in Xinjiang, China. Lancet 344, 107–110 10.1016/S0140-6736(94)91286-6 7912349

[B4] De Benoist B. Andersson M. Egli I. Takkouche B. Allen H. (Eds). (2004). Iodine status worldwide, in WHO Global Database on Iodine Deficiency, (Geneva: World Health Organization), 12–13

[B5] Hong C. L. Weng H. X. Yan A. L. Islam E. U. (2009). The fate of exogenous iodine in pot soil cultivated with vegetables. Environ. Geochem. Health 31, 99–108 10.1007/s10653-008-9169-6 18386132

[B6] Mackowiak C. L. Grossl P. R. (1999). Iodate and iodide effects on iodine uptake and partitioning in rice (*Oryza sativa* L.) grown in solution culture. Plant Soil 212, 135–143 10.1023/A:1004666607330 11762382

[B7] Muramatsu Y. Christoffers D. Ohmomo Y. (1983). Influence of chemical forms on iodine uptake by plant. J. Radiat. Res. 24, 326–338 10.1269/jrr.24.326 6676469

[B8] Muramatsu Y. Uchida S. Sumiya M. Ohmomo Y. Obata H. (1989). Tracer experiments on transfer of radio-iodine in the soil-rice plant system. Water Air Soil Pollut. 45, 157–171 10.1007/BF00208585

[B9] Tagami K. Uchida S. Hirai I. Tsukada H. Takeda H. (2006). Determination of chlorine, bromine and iodine in plant samples by inductively coupled plasma-mass spectrometry after leaching with tetramethyl ammonium hydroxide under a mild temperature condition. Anal. Chim. Acta 570, 88–92 10.1016/j.aca.2006.04.011

[B10] Tensho K. (1970). lodine and bromine in soil-plant system with special reference to “Reclamation-Akagare Disease” of lowland rice. Jpn. Agric. Res. Q. 5, 26–32

[B11] Umaly R. C. Poel L. W. (1971). Effects of iodine in various formulations on the growth of barley and pea plants in nutrient solution culture. Ann. Bot. 35, 127–131

[B12] Voogt W. Holwerda H. T. Khodabaks R. (2010). Biofortification of lettuce (*Lactuca sativa* L.) with iodine: the effect of iodine form and concentration in the nutrient solution on growth, development and iodine uptake of lettuce grown in water culture. J. Sci. Food Agric. 90, 906–913 10.1002/jsfa.3902 20355129

[B13] Watanabe I. Tensho K. (1970). Further study on iodine toxicity in relation to “Reclamation Akagare” disease of lowland rice. Soil Sci. Plant Nutr. 16, 192–194 10.1080/00380768.1970.10432839

[B14] Weng H. X. Hong C. L. Yan A. L. Pan L. H. Qin Y. C. Bao L. T. (2008a). Mechanism of iodine uptake by cabbage: effects of iodine species and where it is stored. Biol. Trace Elem. Res. 125, 59–71 10.1007/s12011-008-8155-2 18521548

[B15] Weng H. X. Weng J. K. Yan A. L. Hong C. L. Yong W. B. Qin Y. C. (2008b). Increment of iodine content in vegetable plants by applying iodized fertilizer and the residual characteristics of iodine in soil. Biol. Trace Elem. Res. 123, 218–228 10.1007/s12011-008-8094-y 18265951

[B16] Yamada H. Onagawa Y. Adachi T. Takahashi K. Yonebayashi K. (2006). Relationship between soil iodine and “Akagare” disease of rice plant. Jpn. J. Soil Sci. Plant Nutr. 77, 563-568

[B17] Yamada H. Takeda C. Mizushima A. Yoshino K. Yonebayashi K. (2005). Effect of oxidizing power of roots on iodine uptake by rice plants. Soil Sci. Plant Nutr. 51, 141–145 10.1111/j.1747-0765.2005.tb00018.x

[B18] Yonehara N. Kozono S. Sakamoto H. (1991). Flow injection-spectrophotometric determination of trace amounts of iodide by its catalytic effect on the 4, 4'-bis (dimethylamino)-diphenylmethane-Chloramine T reaction. Anal. Sci. 7, 229–234

[B19] Yoshida S. Muramatsu Y. Katou S. Sekimoto H. (2007). Determination of the chemical forms of iodine with IC-ICP-MS and its application to environmental samples. J. Radioanal. Nucl. Chem. 273, 211–214 10.1007/s10967-007-0738-4

[B20] Yuita K. (1992). Dynamics of iodine, bromine, and chlorine in soil, II: chemical forms of iodine in soil solutions. Soil Sci. Plant Nutr. 38, 281–287 10.1080/00380768.1992.10416491

[B21] Zhu Y. G. Huang Y. Z. Hu Y. Liu Y. X. (2003). Iodine uptake by spinach (*Spinacia oleracea* L.) plants grown in solution culture: effects of iodine species and solution concentrations. Environ. Int. 29, 33–37 10.1016/S0160-4120(02)00129-0 12605934

